# Rate of decline in kidney function and known age-of-onset or duration of type 2 diabetes

**DOI:** 10.1038/s41598-021-94099-3

**Published:** 2021-07-19

**Authors:** Oyunchimeg Buyadaa, Agus Salim, Jedidiah I. Morton, Dianna J. Magliano, Jonathan E. Shaw

**Affiliations:** 1 Department of Clinical Diabetes and Epidemiology, Baker Heart and Diabetes Institute, 99 Commercial Road, Melbourne, VIC 3004 Australia; 2 School of Public Health and Preventive Medicine, Monash University, Melbourne, Australia; 3 Baker Heart and Diabetes Institute, Melbourne, Australia; 4 Centre for Epidemiology and Biostatistics, Melbourne School of Population and Global Health, The University of Melbourne, Melbourne, Australia; 5 School of Mathematics and Statistics, The University of Melbourne, Melbourne, Australia; 6 Department of Mathematics and Biostatistics, La Trobe University, Melbourne, Australia

**Keywords:** Diseases, Endocrinology

## Abstract

The association between rate of kidney function decline and age-of-onset or duration of diabetes has not been well investigated. We aimed to examine whether rates of estimated glomerular filtration rate (eGFR) decline differ by age-of-onset or duration in people with type 2 diabetes. Using the Action to Control Cardiovascular Risk in Diabetes study which included those with HbA1c ≥ 7.5% and who were at high risk of cardiovascular events,, rates of eGFR decline were calculated and were compared among groups defined by the known age-of-onset (0–39, 40–49, 50–59, 60–69 and > 70 years) and 5-year diabetes duration intervals. Changes in renal function were evaluated using median of 6 (interquartile range 3–10) eGFR measurements per person. eGFR decline was the slowest in those with known age-at-diagnosis of 50–59 years or those with duration of diabetes < 5 years. The rates of eGFR decline were significantly greater in those with known age-of-onset < 40 years or those with duration of diabetes > 20 years compared to those diagnosed at 50–59 or those with duration of diabetes < 5 years (− 1.98 vs − 1.61 mL/min/year; − 1.82 vs − 1.52 mL/min/year; respectively (p < 0.001). Those with youngest age-of-onset or longer duration of diabetes had more rapid declines in eGFR compared to those diagnosed at middle age or those with shorter duration of diabetes.

## Introduction

Type 2 diabetes diagnosed at a younger age is reported to run an aggressive course and have higher rates of complications compared to older onset type 2 diabetes^1–4^. Indeed, a number of studies have shown that the risk of end-stage kidney disease (ESKD) is higher in younger-onset (i.e., age-of-onset < 40 years) type 2 diabetes than in older-onset (i.e., age-of-onset ≥ 40 years) type 2 diabetes, and this excess risk is primarily attributable to attainment of a longer duration of diabetes^2,5,6^. This would suggest that for renal outcomes, younger onset type 2 diabetes is not inherently more aggressive than older onset type 2 diabetes. However, results of these studies of ESKD may be limited, as those younger at onset of diabetes will typically need to have had a greater decline in renal function to reach the ESKD endpoint, as their estimated glomerular filtration rate (eGFR) is typically higher when diabetes is diagnosed compared to their older counterparts. Thus, similar incidence of ESKD may co-exist with different rates of decline in renal function. To gain further understanding of any differences in pathophysiology between younger and older onset or those with shorter and longer duration of type 2 diabetes, analyses of changes in kidney function over time should also be examined. The aim of this study, therefore, was to examine whether rates of eGFR decline differ by known age-of-onset or duration in people with type 2 diabetes. The Action to Control Cardiovascular Risk in Diabetes (ACCORD) clinical trial dataset provides a large sample size with multiple measures of eGFR over time, allowing this aim to be comprehensively explored.

## Methods

### Study design and participants

Detailed information about the ACCORD study is described elsewhere^7^. In brief, ACCORD was a multicentre randomised clinical trial in the US and Canada, comprising 10,251 people with type 2 diabetes, glycated haemoglobin levels (HbA1c) of 7.5% or more, aged 40–79 with a history of cardiovascular disease (CVD) or the presence of risk factors for CVD. ACCORD recruited participants between 2003 and 2005. People with type 1 diabetes were excluded via history and clinical assessment. The passive ACCORD Follow-On (ACCORDION) study involved observation of those members of the ACCORD population who agreed to participate for long-term follow-up. The ACCORD study was approved by the Institutional Review Boards of each study centres and adhered to the principles of the Declaration of Helsinki. All study participants provided written informed consent forms^8^. For the current analysis, we included 9917 participants after excluding those with missing diabetes duration (n = 92), missing baseline eGFR (n = 59) or absence of any eGFR measurements after baseline (n = 183).

### Demographic and clinical variables

Socio-demographics, medical history, concomitant medication use, lifestyle behaviours, health-related quality of life, measures of physical and clinical examinations incorporating kidney function were recorded with different frequency by treatment group assignment as described previously^9^. Medical history including diabetes related data are collected and documented at baseline in the form of a detailed initial medical history and reviewed and recorded at specified follow-up visits.

Serum creatinine was measured at baseline and every 4 months thereafter until the end of the trial and at least once during the post-trial period. The Chronic Kidney Disease Epidemiology Collaboration (CKD-EPI) equation was used to estimate eGFR^10^. Detailed information about measurement methods for laboratory parameters is available in previous reports^7,11^.

### Outcome assessment

In this analysis, we specifically focused on the progression of chronic kidney disease (CKD), and therefore evaluated the annual absolute and percentage changes in eGFR. Rates of eGFR decline were calculated and compared based on the known age-of-onset of diabetes, which was classified into 5 groups: 0–39, 40–49, 50–59, 60–69 and over 70 years and 5-year duration of diabetes intervals^5,12^. ESKD during the trial period is defined as a need for dialysis or renal transplantation or decline of eGFR ≤ 15 mL/min/1.73 m^2^ in the absence of an acute reversible cause. ESKD events during the post-trial period in ACCORDION were not captured. Thus, for the post-trial period, we defined ESKD as progression to a sustained eGFR ≤ 15 mL/min/1.73 m^2^.

### Statistical analyses

Rates of eGFR decline were calculated using a joint longitudinal-survival model and were compared based on the age-of-onset or duration of diabetes^13–15^. In brief, joint-longitudinal survival model is a method that takes into account potentially informative censoring when modelling longitudinal data by simultaneously modelling the longitudinal and survival outcome. This model consists of 2 parts: a model for the trajectory of the longitudinal measurements (eGFR) and a model for the time-to-event (ESKD) data. When longitudinal and event time processes are correlated they reduce the bias obtained from separate a linear mixed effects model or a survival model (time-to-event model). Model 1 included socio-demographic factors; for model 2, smoking status at baseline (yes/no), family history of CVD, personal CVD history, diabetes duration, body mass index, blood pressure levels, use of renin–angiotensin–aldosterone system (RAAS) blockers, glycated haemoglobin, serum lipid levels and urine albumin/creatinine ratio (UACR) were added to model 1. The trajectories of eGFR stratified by age-of-onset of diabetes over time were visualised using duration as the time scale in the analysis. In a sensitivity analysis, we also stratified the analyses by sex. Moreover, in order to determine if hyperfiltration affected the results, we conducted a sensitivity analysis in which only those with a baseline eGFR of less than 120 mL/min/1.73 m^2^ were included, and among whom we estimated rates of eGFR decline using a joint longitudinal-survival model. In a further sensitivity analysis, we restricted the analysis to the trial period in which more frequent creatinine measurements were performed. All statistical analyses were performed in Stata for Windows, version 15 (Stata corporation) and R version 3.6.0 (www.r-project.org).

We received a de-identified dataset from the Biologic Specimen and Data Repository Information Coordinating Center (BioLINCC) after obtaining institutional review board approval from the human research ethics committees of the Alfred Hospital (Project No: 214/18) and Monash University (Project No: 13458), Melbourne, Australia.

### Disclaimer

This study does not necessarily reflect the opinions or reviews of the ACCORD study investigators or NHLBI.

## Results

Tables 1 and 2 show that at baseline, the median age was 62.0 years (interquartile range (IQR) 57.6–67.0), the median eGFR was 87.2 mL/min/1.73 m^2^, and 35.0% had a prior CVD history. The median age at baseline ranged from 58 years (interquartile range (IQR) (55.3–62.6)) in those with known age-of-onset under 40 years, to 76 years (IQR 74.1–78.1) in those with known age-of-onset of over 70 years. The median known diabetes duration ranged from 3.0 (IQR 1.0–5.0) in those with known age-of-onset of over 70 years to 23.0 (IQR 19.0–30.0) in those with known age-of-onset under 40 years (Tables 1 and 2). With regard to kidney function, the median eGFR at baseline was 91 mL/min/1.73 m^2^ in those with known age-of-onset under 40 years and 71 mL/min/1.73 m^2^ in those with known age-of-onset of over 70 years. At baseline, those with known age-of-onset under 40 years or those with known duration of diabetes longer than 20 years had poorer glycaemic control, higher UACR and were more likely to be current smokers. They were more likely to be prescribed RAAS-blockers and insulin compared to other age-of-onset or diabetes duration groups (Table 1). Changes in renal function were evaluated using 108,876 eGFR determinations over 9 years (median: 6 (IQR 3–10) determinations per person). Tables 3 and 4 show the rates of eGFR decline according to known age-of-onset or duration of diabetes. 'When adjusted for baseline age, sex, ethnicity and education (Model 1), those with known age-of-onset of type 2 diabetes under 40 years had a faster decline in eGFR for both absolute and percentage changes compared to those diagnosed at ages 50–59 years. Also, those with known duration of diabetes longer than 20 years had a faster absolute and percentage decline in eGFR compared to those with diabetes duration of less than 5 years. Similarly, the incidence of ESKD was significantly higher in the < 40 age-of-onset group or those with longer diabetes duration (Supplementary Table S1). Those with known age-of-onset over 70 years also had an annual percentage decline in eGFR that was significantly greater than the reference group (− 2.75 vs − 1.99, p < 0.001). Results for Model 2 were similar to those for Model 1.

**Table 1:** Baseline characteristics of study participants according to known age-of-onset of diabetes.

Characteristics	Overall	Known age-of-onset of diabetes (years)				
		0–39	40–49	50–59	60–69	70+
n (%)	9917	945 (9.5)	2926 (29.5)	4264 (43)	1561 (15.8)	221 (2.2)
Baseline age (yr; median [IQR])	62.0 (57.6, 67.0)	58.0 (55.3, 62.6)	58.8 (56.1, 62.8)	61.8 (58.4, 65.7)	69.1 (66.1, 72.4)	76.2 (74.1, 78.1)
Diabetes duration (yr; median [IQR])	9.0 (5.0, 15.0)	23.0 (19.0, 30.0)	13.0 (10.0, 18.0)	7.0 (4.0, 11.0)	5.0 (3.0, 8.0)	3.0 (1.0, 5.0)
Follow-up (yr, mean [SD])	7.0 (2.7)	6.9 (3.1)	7.0 (3.1)	7.1 (3.1)	6.6 (3.0)	5.5 (2.7)
Male (n [%])	6107 (61.6)	542 (57.3)	1815 (62.0)	2633 (61.7)	983 (63.0)	136 (61.5)
**Race (n [%])**						
White	6191 (62.4)	504 (53.3)	1736 (59.3)	2714 (63.7)	1083 (69.4)	154 (69.7)
Black	1883 (19.0)	217 (23.0)	595 (20.3)	773 (18.1)	261 (16.7)	37 (16.7)
Hispanic	709 (7.2)	97 (10.3)	216 (7.4)	311 (7.3)	72 (4.6)	13 (5.9)
Other	1134 (11.4)	127 (13.4)	379 (13.0)	466 (10.9)	145 (9.3)	17 (7.7)
**Education (n [%])**						
< High school	1442 (14.5)	147 (15.5)	391 (13.4)	588 (13.8)	270 (17.3)	46 (20.8)
High school graduate	2620 (26.4)	266 (28.2)	722 (24.7)	1102 (25.8)	466 (29.8)	64 (28.9)
Some college	3257 (32.9)	312 (33.1)	1016 (34.7)	1406 (33.0)	463 (29.7)	60 (27.2)
≥ College graduate	2598 (26.2)	220 (23.2)	797 (27.2)	1167 (27.4)	362 (23.2)	51 (23.1)
Current smoker (n [%])	1375 (13.9)	145 (15.3)	451 (15.4)	594 (13.9)	169 (10.8)	16 (7.2)
HbA1c (%; median [IQR])	8.1 (7.6, 8.9)	8.3 (7.8, 9.1)	8.2 (7.7, 9.0)	8.1 (7.5, 8.8)	7.9 (7.5, 8.6)	7.8 (7.3, 8.6)
HbA1c (mmol/mol; median [IQR])	65.0 (58.5, 72.3)	67.2 (61.7, 75.9)	66.1 (60.6, 74.9)	65.0 (58.5, 72.7)	62.8 (58.5, 70.5)	61.7 (56.3, 70.5)
Insulin use (n [%])	3480 (35.1)	637 (67.4)	1360 (46.5)	1161 (27.2)	298 (19.1)	24 (10.8)
BMI (kg/m^2^; mean [SD])	32.2 (5.4)	32.2 (5.6)	32.5 (5.5)	32.4 (5.3)	31.4 (4.9)	30.2 (4.8)
Triglycerides (mg/dL; median [IQR])	155 (106, 228)	137 (94.5, 202)	151 (102, 232)	160 (112, 236)	157 (109.5, 218.5)	148 (99, 218)
Total cholesterol (mg/dL; median [IQR])	178 (154, 207)	174 (149, 201)	177 (153, 208)	181 (157, 209)	176 (153, 203)	171 (147, 198)
Systolic BP (mmHg; mean [SD])	136.3 (17.0)	136.2 (17.1)	136.1 (16.9)	135.8 (16.8)	137.5 (18.0)	139.0 (16.5)
RAAS blockers (n [%])	6858 (69.1)	698 (73.8)	2102 (71.8)	2864 (67.2)	1039 (66.5)	154 (70.1)
CVD history (n [%])	3472 (35.0)	474 (50.1)	1113 (38.0)	1241 (29.1)	554 (35.5)	90 (40.9)
UACR (mg/g; median [IQR])	14 (7, 44)	20 (8, 86.5)	14 (7, 50)	13 (7, 37)	12 (7, 37)	17 (7, 43)
**UACR categories**						
A1 (normoalbuminuria)	6801 (68.6)	538 (56.9)	1949 (66.6)	3062 (71.8)	1108 (71.0)	144 (65.2)
A2 (microalbuminuria)	2468 (24.9)	297 (31.4)	743 (25.4)	991 (23.2)	371 (23.8)	66 (29.9)
A3 (macroalbuminuria)	648 (6.5)	110 (11.7)	234 (8.0)	211 (5.0)	82 (5.2)	11 (4.9)
eGFR (mL/min/1.73 m^2^; median [IQR])	87.2 (72.2, 96.7)	91.2 (72.7, 99.4)	91.3 (75.6, 99.1)	88.8 (73.1, 96.8)	78.6 (66.7, 90.4)	71.5 (58.6, 83.0)
**eGFR categories (mL/min/1.73 m**^**2**^**)**						
G1 (> 90)	4515 (45.5)	491 (52.0)	1549 (52.9)	2034 (47.7)	422 (27.1)	19 (8.6)
G2 (60–90)	4414 (44.5)	362 (38.3)	1149 (39.3)	1838 (43.1)	923 (59.1)	142 (64.3)
G3 (< 60)	988 (10.0)	92 (9.7)	228 (7.8)	392 (9.2)	216 (13.8)	60 (27.1)

*IQR* interquartile range, *y* year-olds, *yr* year, *HbA1c* glycated haemoglobin, *BMI* body mass index, *BP* blood pressure, *RAAS blockers* renin–angiotensin–aldosterone system blockers, *CVD history* cardiovascular disease history, *UACR* urine albumin-creatinine ratio, *eGFR* estimated glomerular filtration rate.

**Table 2:** Baseline characteristics of study participants according to known duration of diabetes.

Characteristics	Overall	Known duration of diabetes (years)				
		0–4	5–9	10–14	15–19	20+
n (%)	9917	2114 (21.3)	2848 (28.7)	2246 (22.7)	1311 (13.2)	1398 (14.1)
Baseline age (yr; median [IQR])	62.0 (57.6, 67.0)	60.4 (56.8, 65.3)	61.4 (57.4, 66.2)	61.9 (57.7, 66.8)	62.9 (58.5, 68.3)	64.6 (60.1, 70.1)
Age at diagnosis of diabetes (yr; median [IQR])	52.2 (46.2, 58.0)	58.1 (54.3, 62.7)	54.7 (50.8, 59.7)	50.2 (46.3, 55.3)	46.4 (42.0, 51.4)	40.1 (35.0, 45.3)
Follow-up (yr; mean [SD])	6.9 (3.1)	6.9 (3.1)	7.0 (3.1)	7.1 (3.1)	7.0 (3.0)	6.6 (3.1)
Male (n [%])	6107 (61.6)	1294 (61.2)	1772 (62.2)	1383 (61.5)	810 (61.8)	850 (60.8)
**Race (n [%])**						
White	6191 (62.4)	1317 (62.3)	1868 (65.6)	1399 (62.3)	797 (60.8)	810 (57.9)
Black	1883 (19.0)	257 (12.2)	297 (10.4)	251 (11.2)	166 (12.7)	163 (11.7)
Hispanic	709 (7.2)	150 (7.1)	178 (6.3)	179 (7.9)	85 (6.4)	117 (8.4)
Other	1134 (11.4)	390 (18.4)	505 (17.7)	417(18.6)	263 (20.1)	308 (22.0)
**Education (n [%])**						
< High school	1442 (14.5)	274 (12.9)	408 (14.3)	309 (13.8)	184 (14.1)	261 (18.7)
High school graduate	2620 (26.4)	533 (25.2)	740 (26.0)	613 (26.6)	349 (26.6)	385 (27.5)
Some college	3257 (32.9)	727 (34.4)	949 (33.3)	709 (31.6)	414 (31.7)	458 (32.8)
≥ College graduate	2598 (26.2)	580 (27.5)	750 (26.4)	614 (27.3)	361 (27.6)	293 (21.0)
Current smoker (n [%])	1375 (13.9)	341 (16.1)	415 (14.6)	312 (13.9)	151 (11.5)	156 (11.1)
HbA1c (%; median [IQR])	8.1 (7.6, 8.9)	7.9 (7.4, 8.7)	8.1 (7.6, 8.9)	8.2 (7.6, 8.9)	8.1 (7.6, 8.8)	8.2 (7.7, 8.9)
HbA1c (mmol/mol; median [IQR])	65.0 (58.5, 72.3)	62.8 (57.3, 71.5)	65.0 (59.5, 73.7)	66.1 (59.5, 72.6)	65.0 (59.5, 72.6)	66.1 (60.6, 73.7)
Insulin use (n [%])	3480 (35.1)	225 (10.6)	690 (24.2)	908 (40.4)	740 (56.4)	917 (65.6)
BMI (kg/m^2^; mean [SD])	32.2 (5.4)	32.6 (5.4)	32.4 (5.3)	32.2 (5.3)	31.8 (5.4)	31.6 (5.5)
Triglycerides (mg/dL; median [IQR])	155 (106, 228)	166 (118, 246)	165 (114, 237)	152 (105, 229)	137 (96, 205)	133 (94, 199)
Total cholesterol (mg/dL; median [IQR])	178 (154, 207)	185 (159, 213)	179 (156, 208)	177 (154, 207)	174 (151, 202)	171 (149, 199)
Systolic BP (mmHg; mean [SD])	136.3 (17.0)	135.5 (16.2)	135.1 (16.9)	136.5 (17.4)	137.6 (17.4)	138.0 (17.4)
RAAS blockers (n [%])	6858 (69.1)	1299 (61.5)	1954 (68.6)	1592 (70.8)	976 (74.4)	1037 (74.2)
CVD history (n [%])	3472 (35.0)	690 (32.6)	898 (31.5)	764 (34.0)	496 (37.8)	624 (44.6)
UACR (mg/g; median [IQR])	14 (7, 44)	11 (6, 26)	12 (6, 34)	15 (7, 50)	17 (7, 59)	22 (9, 91)
**UACR categories**						
A1 (normoalbuminuria)	6801 (68.6)	1631 (77.2)	2075 (72.8)	1495 (66.6)	823 (62.8)	777 (55.6)
A2 (microalbuminuria)	2468 (24.9)	414 (19.6)	632 (22.2)	591 (26.3)	376 (28.7)	455 (32.6)
A3 (macroalbuminuria)	648 (6.5)	69 (3.2)	141 (5.0)	159 (7.1)	112 (8.5)	166 (11.8)
eGFR (mL/min/1.73 m^2^; median [IQR])	87.2 (72.2, 96.7)	91.2 (77.7, 99.1)	88.7 (73.5, 97.4)	86.7 (72.1, 96.7)	83.7 (69.5, 94.8)	79.6 (65.2, 93.3)
**eGFR categories (mL/min/1.73 m**^**2**^**)**						
G1 (> 90)	4515 (45.5)	1134 (53.6)	1356 (47.7)	1026 (45.7)	528 (40.3)	471 (33.7)
G2 (60–90)	4414 (44.5)	852 (40.3)	1237 (43.4)	1103 (45.1)	618 (41.1)	694 (49.6)
G3 (< 60)	988 (10.0)	128 (6.1)	255 (8.9)	207 (9.2)	165 (12.6)	233 (16.7)

*IQR* interquartile range, *y* year-olds, *yr* year, *HbA1c* glycated haemoglobin, *BMI* body mass index, *BP* blood pressure, *RAAS blockers* renin–angiotensin–aldosterone system blockers, *CVD history* cardiovascular disease history, *UACR* urine albumin-creatinine ratio, *eGFR* estimated glomerular filtration rate.

**Table 3:** Annual change in eGFR according to known age-of-onset of diabetes.

	N	Absolute eGFR change (mL/min/1.73 m^2^ per yr)^a^	Difference in absolute eGFR change (95% CI)	Percentage eGFR change (% per yr)^a^	Difference in percentage eGFR change (95% CI)
**Model 1**					
0–39 y	945	− 2.01 (2.45)	− 0.38 (− 0.58, − 0.17)	− 2.46 (2.64)	− 0.47 (− 0.65, − 0.29)
40–49 y	2926	− 1.78 (2.04)	− 0.15 (− 0.26, − 0.02)	− 2.13 (2.06)	− 0.12 (− 0.22, − 0.03)
50–59 y	4264	− 1.63 (1.88)	Reference	− 1.99 (1.89)	Reference
60–69 y	1561	− 1.68 (2.35)	− 0.05 (− 0.18, 0.08)	− 2.21 (2.00)	− 0.22 (− 0.33, − 0.10)
≥ 70 y	221	− 1.89 (2.17)	− 0.26 (− 0.61, 0.10)	− 2.75 (2.28)	− 0.76 (− 1.06, − 0.44)
**Model 2**					
0–39 y	943	− 1.98 (2.43)	− 0.37 (− 0.56, − 0.16)	− 2.35 (2.61)	− 0.40 (− 0.57, − 0.22)
40–49 y	2925	− 1.75 (2.06)	− 0.14 (− 0.25, − 0.02)	− 2.05 (2.25)	− 0.10 (− 0.18 0.01)
50–59 y	4261	− 1.61 (1.89)	Reference	− 1.95 (1.94)	Reference
60–69 y	1559	− 1.62 (1.88)	− 0.01 (− 0.14, 0.13)	− 2.06 (2.08)	− 0.11 (− 0.22, 0.01)
≥ 70 y	220	− 1.77 (2.21)	− 0.16 (− 0.53, 0.20)	− 2.52 (2.23)	− 0.56 (− 0.91, − 0.21)

Model 1: adjusted for age, sex, race and education. Model 2: Model 1 + smoking status at baseline, family history of cardiovascular disease (CVD), CVD history at baseline, diabetes duration, body mass index, blood pressure levels, use of renin–angiotensin–aldosterone system blockers, glycated haemoglobin level, serum lipid levels, and baseline urine albumin/creatinine, ratio. Baseline eGFR is not included in the covariate set, as it is already present in the joint longitudinal-survival model specification.

*eGFR* estimated glomerular filtration rate, *y* years.

^a^Data are mean (standard deviation).

**Table 4:** Annual change in eGFR according to known duration of diabetes.

	N	Absolute eGFR change (mL/min/1.73 m^2^ per yr)^a^	Difference in absolute eGFR change (95% CI)	Percentage eGFR change (% per yr)^a^	Difference in percentage eGFR change (95% CI)
**Model 1**					
0**–**4 y	2114	− 1.55 (1.87)	Reference	− 1.83 (1.81)	Reference
5**–**9 y	2848	− 1.65 (1.94)	− 0.10 (− 0.23, 0.03)	− 2.00 (1.94)	− 0.17 (− 0.28, − 0.07)
10**–**14 y	2246	− 1.80 (1.91)	− 0.25 (− 0.39, − 0.11)	− 2.20 (1.93)	− 0.37 (− 0.49, − 0.26)
15**–**19 y	1311	− 1.82 (2.18)	− 0.27 (− 0.44, − 0.09)	− 2.31 (2.35)	− 0.48 (− 0.62, − 0.33)
≥ 20 y	1398	− 1.90 (2.17)	− 0.35 (− 0.52, − 0.17)	− 2.53 (2.41)	− 0.70 (− 0.85, − 0.55)
**Model 2**					
0**–**4 y	2110	− 1.52 (1.81)	Reference	− 1.82 (1.74)	Reference
5**–**9 y	2832	− 1.62 (1.89)	− 0.10 (− 0.22, 0.04)	− 1.91 (1.89)	− 0.09 (− 0.19, 0.01)
10**–**14 y	2236	− 1.77 (1.92)	− 0.25 (− 0.38, − 0.11)	− 2.16 (1.95)	− 0.34 (− 0.45, − 0.23)
15**–**19 y	1307	− 1.76 (2.14)	− 0.40 (− 0.40, − 0.06)	− 2.21 (2.31)	− 0.39 (− 0.54, − 0.25)
≥ 20 y	1393	− 1.82 (2.17)	− 0.30 (− 0.46, − 0.12)	− 2.40 (2.39)	− 0.58 (− 0.72, − 0.43)

Model 1: adjusted for age, sex, race and education. Model 2: Model 1 + smoking status at baseline, family history of cardiovascular disease (CVD), CVD history at baseline, diabetes duration, body mass index, blood pressure levels, use of renin–angiotensin–aldosterone system blockers, glycated haemoglobin level, serum lipid levels, and baseline urine albumin/creatinine, ratio. Baseline eGFR is not included in the covariate set, as it is already present in the joint longitudinal-survival model specification.

*eGFR* estimated glomerular filtration rate, *y* years.

^a^Data are mean (standard deviation).

**Figure 1:**
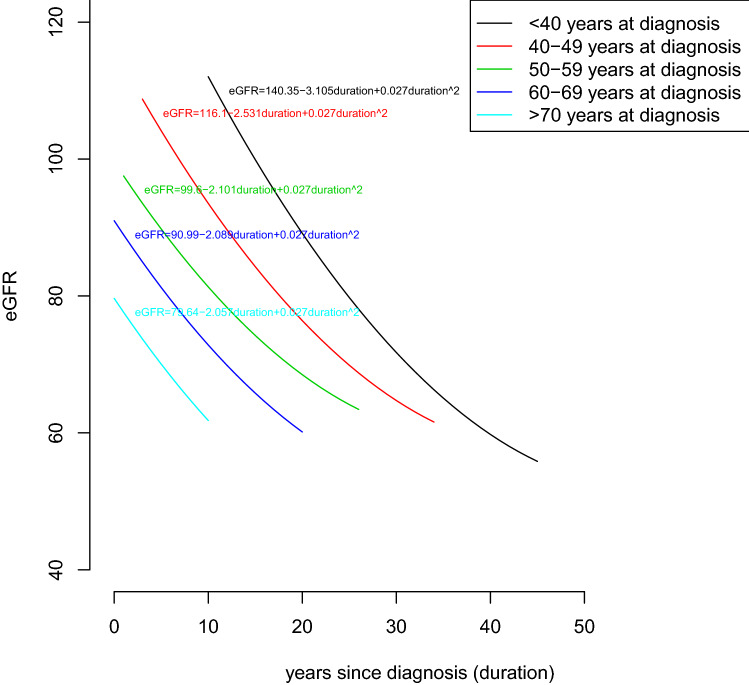
Estimated trajectories of log-transformed estimated glomerular filtration rate (eGFR) levels according to known diabetes duration based on Model 1, stratified by known age-of-onset of diabetes.

Figure 1 shows the trajectories of eGFR stratified by age-of-onset of diabetes. Ten years after diagnosis, people with known age-of-onset of diabetes under 40 years had the highest average eGFR. However, they experience the most rapid decline in kidney function compared to those diagnosed later in life. Over a 10-year period (e.g. from 15 to 25 years duration), their average eGFR declines by 10 mL/min/1.73 m^2^ [95% confidence interval 8.0–12.1] more than does the eGFR of those diagnosed at age 50–59 years.

### Sensitivity analyses

In a separate analysis, we compared the rate of decline in kidney function according to known age-of-onset or duration of diabetes, stratified by sex. The patterns of the rate of eGFR decline were similar to that of our main analysis (Supplementary Table S2). Both absolute and percentage changes in eGFR were similar to the main results when we excluded those with eGFR > 120 mL/min/1.73 m^2^ (Supplementary Table S3). Results were similar in another sensitivity analysis restricted to the trial period, except that those with known age-of-onset over 70 years had a lower annual absolute, but not percentage, decline in eGFR compared to the reference group (Supplementary Table S4).

## Discussion

Using data from this prospective cohort study, we showed that rates of eGFR decline are faster in the youngest age of type 2 diabetes onset group and in those with longest duration of diabetes compared to those with onset aged 50–59 years or those with duration of diabetes less than 5 years, respectively. We further showed that in this cohort of people with type 2 diabetes, among those with similar but longer diabetes durations, the rate of eGFR decline is greatest in those with earliest diabetes onset.

The baseline clinical characteristics of study participants with younger onset (those with known age-of-onset < 40 years) or longer duration (those with known diabetes duration > 20 years) of type 2 diabetes in this study were similar to those reported in other studies^16,17^. The younger diabetes onset or those with longer duration group had worse glycaemic control and were more likely to be current smokers. These unfavourable risk factors may partly contribute to a more aggressive progression of diabetes and higher rates of complications. In addition, the prevalence of albuminuria was higher in those with younger-onset or longer duration of diabetes compared to any other group. These results suggest that albuminuria could be more relevant for kidney dysfunction in type 2 diabetes. This is in keeping our previous analysis of the ACCORD cohort^15^.

While there is good evidence showing that younger onset type 2 diabetes confers an increased risk of ESKD^2,4^, a finding we confirmed here, these studies do not report on rates of eGFR decline by age-of-onset or duration of diabetes. Thus, this may not represent differences in pathophysiology of CKD progression between younger and older onset type 2 diabetes or how pathophysiology changes with increasing diabetes duration. Clearly, individuals who develop diabetes at a younger age will need to have had a greater decline in renal function to reach ESKD, because their initial eGFR is typically higher when diabetes is diagnosed^2,16,18^. Examination of the rate of decline in eGFR therefore provides important additional insights into the impact of younger onset or longer duration of type 2 diabetes. Our current analyses of changes in eGFR over time demonstrated that both the mean absolute and percentage annual declines in eGFR in people with younger onset type 2 diabetes appears to be greater than those diagnosed in middle age, and that the annual eGFR decline increases with increasing duration of diabetes. These findings suggest that the pathophysiological mechanisms of CKD progression in younger onset diabetes may be different to older onset diabetes; and that known age-of-onset and duration of diabetes are both important factors underlying the progression of CKD in diabetes.

There are several possible explanations for our findings. One possible explanation for rapid decline in eGFR in people with younger onset (those with known age-of-onset < 40 years) type 2 diabetes, is the hyperfiltration and its subsequent normalisation in this group, which may lead to greater decline in eGFR^8,19^. However, this explanation is unlikely, because differences in eGFR decline persisted after excluding those with possible hyperfiltration in our sensitivity analysis. It is possible that some features of obesity, including increased levels of fatty acids and leptin, may contribute to greater decline in kidney function in those who develop diabetes at younger age^20,21^. The elevated levels of fatty acids is accompanied by increased oxidative stress which, together may result in premature kidney damage^22^. The biomarkers associated with progression of kidney disease may also change with increasing diabetes duration. Furthermore, genetic predisposition and other unknown factors associated with a younger onset of type 2 diabetes may play a role in the more rapid decline in kidney function in this group^23,24^.

Although people with an age-of-onset over 70 years seemed to have a greater decline in eGFR compared to the reference group in the primary analysis, when we restricted the analysis to the ACCORD trial period, this was no longer statistically significant. It is not clear why this only affected the oldest age-of-onset group. It is possible that the intensive therapy in the in-trial period was relaxed more quickly at the end of ACCORD in this group than in younger participants. Thus, eGFR started to fall more rapidly in the ACCORDION phase.

The strengths of this study are the large sample size, long follow-up and frequent serum creatinine sampling. However, our data should be interpreted carefully in the context of the following limitations. Ascertaining the exact age-of-onset in type 2 diabetes is difficult, because many people remain asymptomatic or undiagnosed for many years. In addition, we were unable to completely account for differences in duration of diabetes between age-of-onset groups. This is because age-of-onset and duration of diabetes are highly correlated, and because of the limited overlap of durations in the younger age-of-onset groups with older age-of-onset. Moreover, what we found mainly applies to people aged over 50 years old, and cannot necessarily be extrapolated to the earlier years of diabetes in those with younger onset type 2 diabetes. Lastly, the generalizability of our findings may be limited to some extent, because the population was drawn from a clinical trial that recruited those with HbA1c ≥ 7.5% and who were at high risk of CVD events. However, we believe that this cohort represents a diverse group of people with type 2 diabetes and our findings add significantly to the understanding of potential differences in progression of CKD for those with a younger onset of type 2 diabetes.

## Conclusion

Our current study suggests that in cohort of people type 2 diabetes with HbA1c ≥ 7.5% and who were at high risk of CVD events, those with younger age-of-onset or longer duration of type 2 diabetes may have a more rapid decline in eGFR compared to those diagnosed in middle age or those with shorter duration of diabetes. These findings contribute to the body of evidence suggesting that early and careful monitoring of kidney function is warranted in those with younger onset type 2 diabetes as they are at the highest long-term risk for kidney complications. Interventions which halt or slow the decline of eGFR are needed in this group.

## Supplementary Information

Supplementary Tables.
