# Supplementary material for: Rate of decline in kidney function and known age-of-onset or duration of type 2 diabetes
Source: Sci Rep. 2021 Jul 19;11:14705. doi: 10.1038/s41598-021-94099-3 (PMC8290031; doi:10.1038/s41598-021-94099-3)
Supplement: Supplementary file 1 — Supplementary Tables. [file 41598_2021_94099_MOESM1_ESM.pdf]

Rate of Decline in Kidney Function and Known Age-of-Onset or Duration of Type 2 Diabetes

Oyunchimeg Buyadaa[1, 2], Agus Salim[3, 4], Jedidiah I Morton[1, 2], Dianna J Magliano[1, 2*], and Jonathan E Shaw[1, 2*]

Supplementary material

Contents

Table S1. Incidence of ESKD and hazard ratio estimates from a joint-longitudinal survival model during the overall study and trial periods according to known age-of-onset or duration of diabetes.

Table S2. Annual change in eGFR according to known age-of-onset or duration of diabetes based on Model 2, stratified by sex

Table S3. Annual change in eGFR according to know age-of-onset or duration of diabetes among those with an eGFR less than 120ml/min/1.73 m^2 at baseline.

Table S4. Annual change in eGFR during the trial period according known age-of-onset or duration of diabetes.

Table S1. Incidence of ESKD and hazard ratio estimates from a joint-longitudinal survival model during the overall study and trial periods according to known age-of-onset or duration of diabetes

	ESKD events	Person-years	Rates (95% CI)[a]	Hazard ratio (95% CI)[b]
**Known age-of-onset of diabetes**				
**Overall study period**				
0–39 y	46	8346	5.51 (4.12, 7.36)	1.49 (1.15, 1.82)
40–49 y	101	26716	3.78 (3.11, 4.59)	1.12 (0.87, 1.38)
50–59 y	133	38864	3.42 (2.88, 4.05)	1.00 (Reference)
60–69 y	40	13817	2.89 (2.12, 3.94)	0.80 (0.56, 1.16)
≥70 y	3	1782	1.68 (0.54, 5.22)	0.26 (0.11, 1.41)
**Trial period**				
0–39 y	40	4506	8.87 (6.51, 12.10)	1.33 (0.99, 1.69)
40–49 y	86	14298	6.01 (4.86, 7.43)	0.98 (0.71, 1.24)
50–59 y	121	20869	5.79 (4.85, 6.92)	1.00 (Reference)
60–69 y	37	7627	4.85 (3.51, 6.69)	0.98 (0.60, 1.35)
≥70 y	3	1074	2.79 (0.90, 8.65)	0.61 (0.46, 1.76)
**Known duration of diabetes**				
**Overall study period**				
0–4 y	53	14741	3.59 (2.74, 4.70)	1.00 (reference)
5–9 y	95	20034	4.74 (3.87, 5.79)	1.28 0.95, 1.61)
10–14 y	60	16021	3.74 (2.90, 4.82)	1.06 (0.70, 1.42)
15–19 y	53	9297	5.70 (4.35, 7.46)	1.52 (1.14, 1.91)
≥20 y	59	9331	6.32 (4.89, 8.16)	1.68 (1.31, 2.06)
**Trial period**				
0–4 y	49	13070	3.74 (2.83, 4.96)	Reference
5–9 y	88	17718	4.96 (4.03, 6.12)	1.33 (0.94, 1.89)
10–14 y	48	14144	3.39 (2.55, 4.50)	0.94 (0.62, 1.38)
15–19 y	48	8249	5.81 (4.38, 7.72)	1.63 (1.09, 2.44)
≥20 y	54	8368	6.45 (4.94, 8.42)	1.76 (1.19, 2.62)

a: Rates per 1000 person-years

b: Adjusted for age, sex, race and education.

Abbreviations: ESKD, end-stage kidney disease; y, year-olds.

Table S2. Annual change in eGFR according to known age-of-onset or duration of diabetes based on Model 2, stratified by sex

	**Male**					**Female**				
	N	Absolute eGFR change (mL/min/1.73 m^2 per yr)[a]	Difference in absolute eGFR change (95% CI)	Percentage eGFR change (% per yr)[a]	Difference in percentage eGFR change (95% CI)	N	Absolute eGFR change (mL/min/1.73 m^2 per yr)[a]	Difference in absolute eGFR change (95% CI)	Percentage eGFR change (% per yr)[a]	Difference in percentage eGFR change (95% CI)
**Known age-of-onset of diabetes**										
0–39 y	541	-1.84 (2.29)	-0.27 (-0.52, -0.03)	-2.17 (2.34)	-0.28 (-0.49, -0.08)	401	-2.13 (2.49)	-0.47 (-0.79, -0.15)	-2.68 (2.76)	-0.64 (-0.92, -0.35)
40–49 y	1813	-1.66 (2.06)	-0.09 (-0.23, 0.06)	-1.94 (2.05)	-0.05 (-0.17, 0.06)	1107	-1.87 (1.96)	-0.21 (-0.40, -0.03)	-2.26 (2.02)	-0.22 (-0.36, -0.06)
50–59 y	2623	-1.57 (1.84)	- Reference	-1.89 (1.86)	Reference	1621	-1.66 (1.85)	Reference	-2.04 (1.85)	Reference
60–69 y	980	-1.58 (1.76)	-0.01 (-0.17, 0.15)	-2.01 (1.86)	-0.12 (-0.25, 0.01)	575	-1.69 (1.92)	-0.03 (-0.26, 0.19)	-2.26 (2.12)	-0.22 (-0.41, -0.02)
≥70 y	136	-1.79 (1.87)	-0.22 (-0.54, 0.11)	-2.53 (2.69)	-0.64 (-1.10, -0.18)	84	-1.74 (1.70)	-0.08 (-0.45, 0.30)	-2.49 (2.46)	-0.45 (-0.98. 0.08)
**Known duration of diabetes**										
0–4 y	1294	-1.46 (1.66)	Reference	-1.71 (1.59)	Reference	819	-1.62 (2.03)	Reference	-1.90 (1.93)	Reference
5–9 y	1772	-1.57 (1.90)	-0.11 (-0.27, 0.04)	-1.89 (1.89)	-0.18 (-0.30, 0.05)	1074	-1.68 (1.88)	-0.06 (-0.28, 0.16)	-2.04 (1.89)	-0.14 (-0.31, 0.03)
10–14 y	1382	-1.75 (2.01)	-0.29 (-0.47, -0.12)	-2.11 (2.07)	-0.40 (-0.54, -0.26)	862	-1.81 (1.75)	-0.19 (-0.42, 0.03)	-2.25 (1.75)	-0.35 (-0.53, -0.17)
15–19 y	810	-1.64 (2.16)	-0.18 (-0.39, 0.03)	-2.01 (2.28)	-0.30 (-0.47, -0.11)	501	-1.96 (2.09)	-0.34 (-0.62, -0.04)	-2.54 (2.34)	-0.64 (-0.89, -0.40)
≥20 y	849	-1.71 (2.10)	-0.25 (-0.46, -0.03)	-2.20 (2.22)	-0.49 (-0.6, -0.31)	548	-1.98 (2.24)	-0.36 (-0.64, -0.06)	-2.70 (2.61)	-0.80 (-1.05, -0.54)

a: Data are mean (standard deviation).

Adjusted for age, sex, race and education, smoking status at baseline, family history of cardiovascular disease (CVD), CVD history at baseline, diabetes duration, body mass index, blood pressure levels, use of renin-angiotensin-aldosterone system blockers, glycated haemoglobin level, serum lipid levels, and baseline urine albumin/creatinine, ratio. Baseline eGFR is not included in the covariate set, as it is already present in the joint longitudinal-survival model specification.

Abbreviations: eGFR, estimated glomerular filtration rate; y, years

Table S3. Annual change in eGFR according to known age-of-onset or duration of diabetes among those with an eGFR less than 120 ml/min/1.73 m^2 at baseline

	N	Absolute eGFR change (mL/min/1.73 m^2 per yr)[a]	**Difference in absolute eGFR change (95% CI)**	Percentage eGFR change (% per yr)[a]	**Difference in percentage eGFR change (95% CI)**
**Known age-of-onset of diabetes**					
**Model 1**					
0–39 y	932	-2.02 (2.46)	-0.39 (-0.59, -0.18)	-2.48 (2.66)	-0.49 (-0.67, -0.31)
40–49 y	2889	-1.77 (2.05)	-0.14 (-0.25, -0.02)	-2.12 (2.08)	-0.13 (-0.22, -0.03)
50–59 y	4244	-1.63 (1.88)	Reference	-1.99 (1.90)	Reference
60–69 y	1559	-1.68 (1.86)	-0.05 (-0.18, 0.08)	-2.21 (2.25)	-0.22 (-0.34, -0.08)
≥70 y	220	-1.88 (2.00)	-0.25 (-0.61, 0.09)	-2.75 (2.29)	-0.76 (-1.06, -0.44)
**Model 2**					
0–39 y	932	-1.99 (2.45)	-0.38 (-0.58, -0.17)	-2.37 (2.64)	-0.44 (-0.62, -0.26)
40–49 y	2889	-1.74 (2.06)	-0.14 (-0.25, -0.01)	-2.05 (2.12)	-0.12 (-0.21, -0.02)
50–59 y	4244	-1.60 (1.89)	Reference	-1.93 (1.95)	Reference
60–69 y	1559	-1.61 (1.88)	-0.01 (-0.14, 0.12)	-2.09 (2.08)	-0.15 (-0.27, -0.03)
≥70 y	220	-1.77 (2.15)	-0.17 (-0.53, 0.20)	-2.55 (2.56)	-0.62 (-0.96, -0.27)
**Known duration of diabetes**					
**Model 1**					
0–4 y	2096	-1.80 (2.27)	Reference	-2.12 (2.20)	Reference
5–9 y	2828	-1.97 (2.24)	-0.17 (-0.33, -0.01)	-2.40 (2.21)	-0.28 (-0.40, -0.15)
10–14 y	2227	-2.14 (2.26)	-0.33 (-0.51, -0.16)	-2.64 (2.27)	-0.51 (-0.64, -0.38)
15–19 y	1304	-2.19 (2.61)	-0.39 (-0.60, -0.17)	-2.76 (2.78)	-0.63 (-0.81, -0.46)
≥20 y	1395	-2.24 (2.60)	-0.44 (-0.65, -0.22)	-2.96 (2.84)	-0.84 (-1.01, -0.66)
**Model 2**					
0–4 y	2092	-1.66 (2.21)	Reference	-1.94 (2.12)	Reference
5–9 y	2815	-1.79 (2.20)	-0.13 (0.29, 0.02)	-2.17 (2.17)	-0.22 (-0.34, -0.11)
10–14 y	2218	-1.98 (2.26)	-0.32 (-0.48, -0.15)	-2.43 (2.27)	-0.48 (-0.57, -0.39)
15–19 y	1300	-2.00 (2.56)	-0.34 (-0.55, -0.13)	-2.51 (2.72)	-0.56 (-0.73, -0.39)
≥20 y	1390	-2.04 (2.58)	-0.38 (-0.59, -0.17)	-2.67 (2.79)	-0.72 (-0.89, -0.54)

a: Data are mean (standard deviation).

Model 1: adjusted for age, sex, race and education. **Model 2:** Model 1 + smoking status at baseline, family history of cardiovascular disease (CVD), CVD history at baseline, diabetes duration, body mass index, blood pressure levels, use of renin-angiotensin-aldosterone system blockers, glycated haemoglobin level, serum lipid levels, and baseline urine albumin/creatinine, ratio. Baseline eGFR is not included in the covariate set, as it is already present in the joint longitudinal-survival model specification.

Abbreviations: eGFR, estimated glomerular filtration rate; y, year-olds.

Table S4. Annual change in eGFR during the trial period according to known age-of-onset or duration of diabetes

	N	Absolute eGFR change (mL/min/1.73 m^2 per yr)[a]	Difference in absolute eGFR change (95% CI)	Percentage eGFR change (% per yr)[a]	Difference in percentage eGFR change (95% CI)
**Known age-of-onset of diabetes**					
**Model 1**					
0–39 y	944	-2.43 (2.97)	-0.48 (-0.72, -0.23)	-2.93 (3.13)	-0.53 (-0.74, -0.32)
40–49 y	2926	-2.09 (2.42)	-0.14 (-.27, 0.01)	-2.51 (2.42)	-0.11 (-0.22, 0.001)
50–59 y	4261	-1.95 (2.24)	Reference	-2.40 (2.24)	Reference
60–69 y	1560	-1.98 (2.18)	-0.03 (-0.18, 0.13)	-2.60 (2.33)	-0.20 (-0.33, -0.06)
≥70 y	220	-1.86 (2.11)	0.09 (-0.29, 0.47)	-2.74 (2.40)	-0.34 (-0.66, -0.02)
**Model 2**					
0–39 y	944	-2.26 (2.91)	-0.45 (-0.68,-0.19)	-2.65 (3.14)	-0.47 (-0.68, -0.26)
40–49 y	2926	-1.95 (2.44)	-0.14 (-0.27, 0.01)	-2.29 (2.53)	-0.11 (-0.22, 0.005)
50–59 y	4261	-1.81 (2.22)	Reference	-2.18 (2.32)	Reference
60–69 y	1560	-1.82 (2.17)	-0.01 (-0.16, 0.16)	-2.33 (2.44)	-0.15 (-0.29, -0.01)
≥70 y	220	-1.70 (2.22)	0.11 (-0.28, 0.51)	-2.42 (2.71)	-0.24 (-0.61, 0.11)
**Known duration of diabetes**					
**Model 1**					
0–4 y	2114	-1.53 (1.86)	Reference	-1.82 (1.81)	Reference
5–9 y	2848	-1.65 (1.94)	-0.11 (-0.25, 0.01)	-2.01 (1.95)	-0.19 (-0.29, 0.08)
10–14 y	2246	-1.80 (1.91)	-0.27 (-0.41, -0.12)	-2.21 (1.94)	-0.39 (-0.50, -0.27)
15–19 y	1311	-1.82 (2.19)	-0.29 (-0.46, -0.11)	-2.31 (2.36)	-0.49 (-0.64, -0.34)
≥20 y	1398	-1.90 (2.17)	-0.37(-0.54, -0.18)	-2.53 (2.42)	-0.71 (-0.86, -0.56)
**Model 2**					
0–4 y	2110	-1.51 (1.80)	Reference	-1.78 (1.74)	Reference
5–9 y	2832	-1.62 (1.89)	-0.11 (-0.24, 0.02)	-1.95 (1.89)	-0.17 (-0.27, 0.06)
10–14 y	2236	-1.77 (1.92)	-0.26 (-0.40, -0.12)	-2.17 (1.96)	-0.39 (-0.49, -0.27)
15–19 y	1307	-1.76 (2.15)	-0.25 (-0.42, -0.08)	-2.22 (2.32)	-0.44 (-0.58, -0.29)
≥20 y	1393	-1.82 (2.17)	-0.31 (-0.48, -0.14)	-2.40 (2.40)	-0.62 (-0.76, -0.47)

a: Data are mean (standard deviation).

Model 1: adjusted for age, sex, race and education. Model 2: Model 1 + smoking status at baseline, family history of cardiovascular disease (CVD), CVD history at baseline, diabetes duration, body mass index, blood pressure levels, use of renin-angiotensin-aldosterone system blockers, glycated haemoglobin level, serum lipid levels, and baseline urine albumin/creatinine, ratio. Baseline eGFR is not included in the covariate set, as it is already present in joint longitudinal-survival model specification.

Abbreviations: eGFR, estimated glomerular filtration rate; y, years.
